# CGG toolkit: Software components for computational genomics

**DOI:** 10.1371/journal.pcbi.1011498

**Published:** 2023-11-07

**Authors:** Dimitrios Vasileiou, Christos Karapiperis, Ismini Baltsavia, Anastasia Chasapi, Dag Ahrén, Paul J. Janssen, Ioannis Iliopoulos, Vasilis J. Promponas, Anton J. Enright, Christos A. Ouzounis

**Affiliations:** 1 Biological Computation & Process Laboratory, Chemical Process & Energy Resources Institute, Centre for Research & Technology Hellas, Thessalonica, Greece; 2 Biological Computation & Computational Biology Group, AIIA Lab, School of Informatics, Aristotle University of Thessalonica, Thessalonica, Greece; 3 Computational Biology Group, Faculty of Medicine, University of Crete, Heraklion, Greece; 4 Department of Biology, Microbial Ecology Group, Lund University, Lund, Sweden; 5 Nuclear Medical Applications, Belgian Nuclear Research Centre SCK CEN, Mol, Belgium; 6 Bioinformatics Research Laboratory, Department of Biological Sciences, New Campus, University of Cyprus, Nicosia, Cyprus; 7 Department of Pathology, University of Cambridge, Tennis Court Road, Cambridge, United Kingdom; 8 SysBioBio.info (SBBI), Thessalonica, Greece; Universite de Lausanne Faculte de biologie et medecine, SWITZERLAND

## Abstract

Public-domain availability for bioinformatics software resources is a key requirement that ensures long-term permanence and methodological reproducibility for research and development across the life sciences. These issues are particularly critical for widely used, efficient, and well-proven methods, especially those developed in research settings that often face funding discontinuities. We re-launch a range of established software components for computational genomics, as legacy version 1.0.1, suitable for sequence matching, masking, searching, clustering and visualization for protein family discovery, annotation and functional characterization on a genome scale. These applications are made available online as open source and include *MagicMatch*, *GeneCAST*, support scripts for *CoGenT*-like sequence collections, *GeneRAGE* and *DifFuse*, supported by centrally administered bioinformatics infrastructure funding. The toolkit may also be conceived as a flexible genome comparison software pipeline that supports research in this domain. We illustrate basic use by examples and pictorial representations of the registered tools, which are further described with appropriate documentation files in the corresponding *GitHub* release.

## Introduction

Genome sequence analysis represents one of the most fundamental elements of computational genomics. It supports structural, comparative and functional genomics, and forms the foundation upon which systematic structure/function prediction, classification and annotation of proteins is based [1]. In addition, it establishes genome-scale properties of species, their relationships and the mapping of encoded genomic components (such as gene loci or protein sequences and structures) to dynamic properties revealed by large-scale genome-scale experiments [2]. Finally, genome sequence analysis is used in taxonomy such as species phylogenies [3], genetics such as protein family discovery [4,5], and biochemistry such as metabolic pathway reconstructions [6].

**Fig 1:**
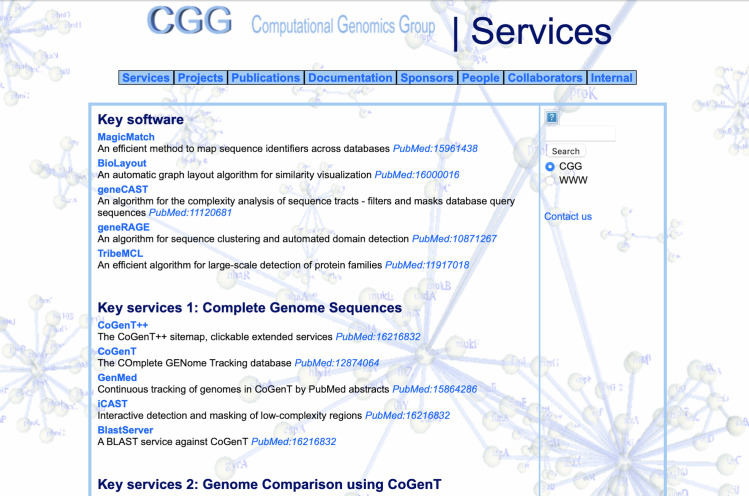
Revived software tools. A 2008 snapshot of the ‘Key software’ section of the *CGG* website followed by services (partly shown), with the list of tools made available again.

In the past, we developed a series of algorithmic components and introduced their software implementations for use in large-scale genome sequence analysis [7]. The Computational Genomics Group (*CGG*) at the European Bioinformatics Institute (1996–2005) maintained a server with these key tools available during the years 1997–2008 (https://web.archive.org/web/*/cgg.ebi.ac.uk) at the *URL* cgg.ebi.ac.uk (aliased as genomes.org), until hardware and other changes forced the discontinuation of these services. In particular, at the *URL* http://cgg.ebi.ac.uk/cgg/Services.html these tools were available until 2008 (Fig 1), with a number of popular software modules either as downloadable source/binary files or as interactive, precomputed solutions (https://web.archive.org/web/20080105003605/http://cgg.ebi.ac.uk/cgg/Services.html). For the following decade or so, every effort had been made to deliver those software components to users by responding to direct requests or maintaining services elsewhere—admittedly a sub-optimal solution, yet the only realistic alternative to ensure public access.

It is widely accepted that most *URL*s published in the recent literature have a limited life-cycle [8], due to discontinued financial support, movement of personnel, inability to scale up resource deployment, and other policy or infrastructure reasons [9]. Unfortunately, the unpredictable and often inadequate grant application review process [10,11] results in the abrupt discontinuation of important bioinformatics services with serious implications for interdisciplinary research [12]. Recent efforts to re-establish and distribute published software in the field have been strongly supported by *ELIXIR*, the principal bioinformatics infrastructure project across Europe [13]. With the creation of funding streams both at the European and national node levels, it has become possible to revive and thus re-distribute previously published services on new platforms not previously available.

Thanks to these developments, we are now re-launching the key software modules of *CGG* services into the public domain that we used in our work for comparative genomics while at the *EBI* and in the ensuing years elsewhere. These exclude pre-computed data collections based on large genomic computations that cannot be made available at present due to the increase in volume of genome information; yet, we illustrate how a number of those data collections might be reconstructed using our toolkit. To that end, wrappers and other control software for managing and integrating various data segments are provided, akin to a flexible computational pipeline that can be modified according to user needs and specifications, thus reviving key components for use by the wider community. This work has been made possible thanks to infrastructure funding by the project ELIXIR-GR.

## Design & implementation

Here, we describe the software tools made available from the angle of usage, and not according to the order in which they were developed or published, guiding users to decide how they deploy the toolkit in various computational genomics projects. A chronological listing is reflected in the original publications (Table 1). All versions have been named v1.0.1, to avoid conflicts with the latest developments and forking of projects in subsequent work. We wish to maintain this version system for consistency and to better plan any software updates with the corresponding funding streams in the near future. We request that citations to software tools refer to both the original publication and this work to acknowledge the re-established availability. We describe the main components below.

**Table 1:** A list of the tools presented and selected, additional work that benefited from them. *Columns*—*GitHub*: name of GitHub repository where the tools and documentation are available (NA: not applicable, as case study)–the prefix of the GitHub folders implies a typical workflow (outlined in Fig 2); *tool*: tool name (or in case of studies, a codeword); *year*: year of original publication; *PMID*: PubMed identifier; *citations*: number of citations reported by Google Scholar on 28-Mar-2023; *citations/yr*: number of citations per year since original publication; *short description*: self-explanatory, for further details, please see original publications. Table is sorted on PMID (which reflects the time of publication).

GitHub	tool	year	PMID	citations	citations/yr	short description
4_generage	DifFuse	1999	10573422	1450	65.91	gene fusion detection
4_generage	GeneRAGE	2000	10871267	276	13.14	clustering & multi-domain detection
1_genecast	GeneCAST	2000	11120681	201	9.57	masking of low-complexity tracts
3_clustt_utils	BioLayout	2001	11590107	208	10.40	network visualization & processing
3_clustt_utils	Tribe-MCL	2002	11917018	3819	201.00	sequence similarity graph clustering
NA-case study	Balance	2003	12840037	237	13.17	balance of forces shaping gene content
NA-case study	GeneTrace	2003	12874054	65	3.61	ancestral gene content reconstruction
2_cogent_utils	CoGenT	2003	12874064	49	2.72	simple identifiers, genome collection
3_clustt_utils	TRIBES	2003	12888524	160	8.89	protein family database
NA-case study	Disease genes	2004	15181176	297	17.47	disease gene profiling
NA-case study	Kingdoms	2005	15681613	72	4.50	genome comparison for taxonomy
0_magicmatch	MagicMatch	2005	15961438	24	1.50	identifier matching protocol
NA-case study	Net of Life	2005	15965028	306	19.13	quantification of gene flow patterns
3_clustt_utils	BioLayout-java	2005	16000016	70	4.38	network visualization & processing
3_clustt_utils	OFAM (CoGent++)	2005	16216832			computed ortholog database
2_cogent_utils	CoGenT++	2005	16216832	27	1.69	computational genomics environment
NA-case study	LUCA estimate	2006	16431085	148	9.87	inference of LUCA’s gene content
		average		sum	average	
		2003		7409	24.18	

MagicMatch is a sequence matching protocol based on the *MD5* checksum for the detection of identical protein sequences [14]. The *MD5* algorithm for message integrity generates fingerprints which are used as hash strings to map sequences across databases. It thus helps the mapping of entry identifiers across sequence collections, which can be a rather time-consuming and computationally complex process. MagicMatch was the first of its kind and follows a minimalist approach. Other, more complex and high-maintenance tools have been proposed [15] compared to which, and for most practical purposes, MagicMatch is superior in speed and usability. An example of use is when whole-genome protein collections are mixed with annotated datasets e.g. *SwissProt* [16] for quick annotation purposes as in the TRIBES database [17]: users will want to find which genome entries are present in the annotated dataset, a task that can be rapidly accomplished using MagicMatch during pre-processing of genome-scale protein sequence datasets.

GeneCAST is a tool for the sensitive detection and selective masking of low-complexity regions in protein sequences [18]. The algorithm is based on multiple-pass Smith-Waterman comparisons [19] of the query sequence against all possible (i.e. 20) homopolymers of amino acid residues with infinite gap penalties. The output generates the masked query sequence that can be used for high-throughput sequence searches with increased sensitivity (fewer false negative hits) and specificity (fewer false positive hits), as well as the statistics and geometry of low-complexity regions.

**Fig 2:**
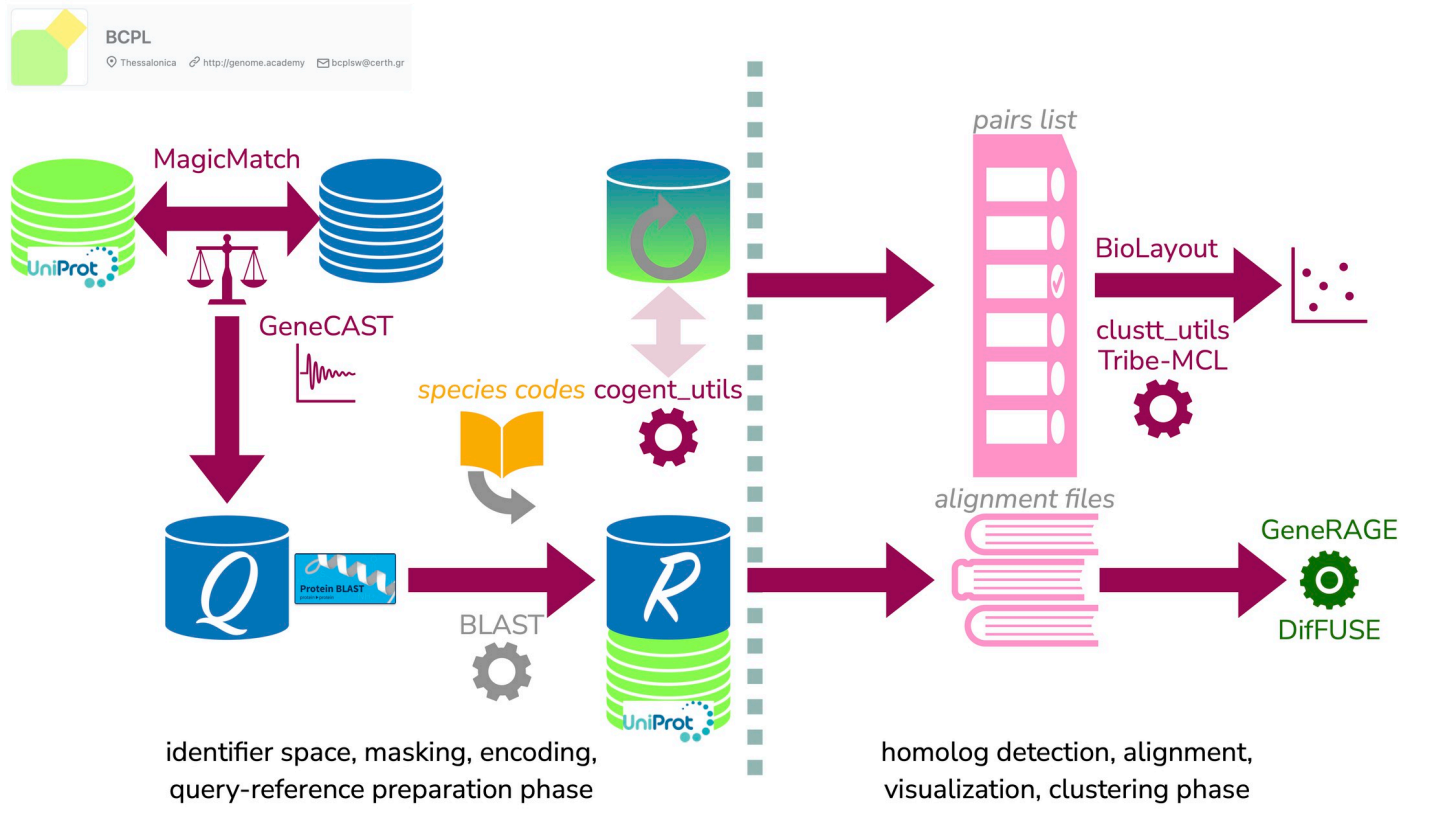
Representation of a typical workflow using the reported tools. Pre-processing may start with a genome collection (database symbol, upper left), optionally mixed with a curated sequence resource such as *UniProt* (database symbol in green, upper left). To cross-index entries at the sequence level or simply identify them, MagicMatch can be used as an option. The sequence collection can be submitted to GeneCAST to mask compositional bias and prepare the query for sensitive searches (disk symbol with Q, lower left). For genome-scale analysis, species codes can be generated for the reference (target) set with cogent_utils, to create a uniformly named sequence set (disk symbol with R, lower middle, optionally mixed with *UniProt* or any other annotated collection). Sequence comparisons are executed with *BLAST* or other options with query Q *vs*. reference R (or in the case of *all-vs-all*, disk symbol in green-blue gradient, upper middle). The vertical gray line divides this pre-processing phase from the next phase, signifying the computationally intensive step or long wall-time. Two (non-mutually exclusive) output alternatives are shown: the pairs-list (in pink, upper right) or full alignments (also in pink, lower right). The former can be treated with clustt_utils that launches Tribe-MCL and generates protein families or can be used as input for network visualization with BioLayout or other similar software, while the latter can be further processed for GeneRAGE or DifFuse for multi-domain or gene-fusion detection, respectively, as well as for inspection and parsing for multiple alignments.

The above two software components are part of the pre-processing steps of the query section for large-scale genome comparisons that typically use as target the entire protein sequence complement encoded in the genome of the corresponding organisms (Fig 2). The next component section refers to the preparation of the target data collection, that generates consistent and tractable sequence identifiers with a few critical annotation strings encoded within a user-defined identifier space. Note that in the case of all-vs-all comparisons, target and query must be identical, a step that is one of the most expensive, computationally demanding parts of genome comparison. An exception to this identity rule might be that the query set is masked by GeneCAST while the target set is not, maintaining the original sequence information, so that targets can be equivalenced back to their source using MagicMatch.

The Complete Genome Tracking (CoGenT) database was originally developed to transform an undisciplined identifier space of genome sequence collections into a highly consistent environment for both human interaction and programming convenience. By using an encoded identifier for genes and species, it aimed at reproducibility, scalability and accessibility [20]. Later, CoGenT was augmented with additional plug-in components as a three-tier system named CoGenT++ [21], where much of the work on the large-scale comparisons of genomic sequences [22], the quantification of gene gain and loss [23], the ancestral reconstructions of gene content [24] and the inference of the gene content of the Last Universal Common Ancestor [25] was based. Despite progress with hardware and software acceleration, CoGenT/CoGenT++ was not extended beyond 250 genomes, when the size of CoGenT++ reached 100 GB in 2006. By 2010, it was one of the few research group-level efforts to keep up with genome catalogs, an objective that is currently achieved only by operations such at the *NCBI* [26] and the *EBI* [27], with varying degrees of success.

To achieve similar functionality, we chose to issue a set of utilities that allow users to recreate the CoGenT style of identifier encodings, named cogent_utils. These utilities are using *shell* scripting with some *awk* and *sed* parts that can read a catalog of genome encodings provided by the user. This action generates a directory with the collections of genomes adopting an encoding scheme comprising the species name, the version (starting with 01) and the incremental numbering of the gene list, so that each gene acquires a unique identifier. Collections can then be concatenated to obtain a full-size CoGenT-like database that may be subsequently indexed for *BLAST* [28] or *DIAMOND* [29,30] searches. The simple yet powerful schema allows the linking of genome sequences to other resources, also facilitated by MagicMatch. A snippet from the *README* file of cogent_utils is provided here, as an illustration.

*Before*: First two sequences headers in file GCF_008822105.2_bTaeGut2.pat.W.v2_protein.faa:

>NP_001041718.1 alpha-synuclein [Taeniopygia guttata]

>NP_001041719.1 neurocalcin-delta [Taeniopygia guttata]

*After*: The first two sequence headers in the generated file Taeg-2p1.faa in the destination folder:

>Taeg-2p1-01-000000 NP_001041718.1 alpha-synuclein [Taeniopygia guttata]

>Taeg-2p1-01-000001 NP_001041719.1 neurocalcin-delta [Taeniopygia guttata]

Once the CoGenT-style sequence collections are processed for database searches and then high-throughput comparisons are executed, the resulting files might optionally generate alignments and/or a pairs-list. The pairs-list (e.g. option 6 for *BLAST*) is an ideal way for summarizing significant hits beyond a certain acceptable threshold value (minimal score or maximal E-value) and can be subjected to visualization and graph clustering. We have implemented a set of utilities as *bash* scripts, named clustt_utils, that capture the output files of large-scale sequence comparison and prepare them for visualization and clustering. For visualization, we primarily use BioLayout, originally developed by the *CGG* [31] and re-implemented in *java* as BioLayout-java [32]. Later, this component was made available at biolayout.org and evolved into *Graphia* [33]. The pairs-list can also be used with other popular platforms such as *Cytoscape* [34]. The script clustt_utils generates pairs-list files as input for BioLayout or *Cytoscape* among others. These lists represent complex sequence similarity graphs that are also used for graph-clustering, where the resulting clusters are interpreted as protein families. Tribe-MCL [35] was the first fully automated approach and the second ever to generate clusters from sequence similarity graphs, a pivotal idea simultaneously proposed by the semi-automated *COG* system [36] around that time. The command line interface of clustt_utils takes as arguments the pairs-list (tabular output of sequence comparisons), the name of the output file, the inflation parameter and the path for *MCL*. This action creates three files, an output file for visualization, the *MCL* output and a human-readable output file with an incremental identifier for families and the sum of members per family for further processing.

As a side-product of the collective effort to revive the *CGG* software, we also release tested versions of the GeneRAGE [37] and DifFuse [38] algorithms, initially implemented to detect multi-domain protein families and gene fusion events, respectively. GeneRAGE was reaching computational bottlenecks for multiple genomes around 2002, a fact that was the trigger for the exploration of other, less computationally demanding algorithms, inspiring early versions of BioLayout [31] and the subsequent adoption of graph-clustering with Tribe-MCL [35]. GeneRAGE builds a binary square matrix and validates non-symmetric relationships using the Smith-Waterman dynamic programming algorithm [19], by either removing false-positive hits or correcting false-negative instances [37]. DifFuse is an analogous implementation, with the difference that the matrix is not square but rectangular, where the shorter dimension represents the ‘query’ species for which gene fusion ‘components’ are requested and the longer dimension represents the ‘reference’ species from which gene fusion ‘composites’ are obtained [38–40]. The clustering results of GeneRAGE and Tribe-MCL can also be compared, as appropriate.

## Results & discussion

The impact of these contributions can be documented directly from the literature, with more than 6000 citations for the tools (6284 on 28-Mar-2023) and an additional 1000 (1125 on 28-Mar-2023) citations for other research by the *CGG* that explicitly used these tools during its existence (Table 1). With an average of ~24 citations/year for 20 years each, this equals to an average of 480 citations per publication, with significant deviations (Tribe-MCL as the most highly cited and MagicMatch and CoGenT++ the least cited, perhaps due to their shorter lifespan and subsequent non-availability). Some of the citing references are heavily cited as well, e.g. *OrthoMCL* [41] or *Roary* [42].

We hope that by making these components accessible again, the expert community will appreciate their merit. We also note that all software can be used without CoGenT identifiers; however, to realize the full power of the suite, it is recommended that CoGenT identifiers are generated. We kindly request that third-party efforts deploying CoGenT-style database creations and comparisons also cite the original papers accordingly.

The tools, source code and usage instructions are available on *BCPL*’s bcpl-certh *GitHub* repository which can be found at https://github.com/bcpl-certh/cgg-toolkit. BioLayout can be downloaded from biolayout.org; it can perform a number of intense computations, including Tribe-MCL types of clustering but can further be used for functional genomics and other visualization activities [43]. All other tools are terminal-based and require a command *shell*, preferably *bash*. The main advantage of *bash* is its cross-platform support and the ease and flexibility to design custom behavior. By utilizing custom *bash* programming, users are able to reconfigure the current toolkit, automate it and extend it according to their needs.

The suite of tools presented herein facilitates large-scale genomic comparisons with attested quality, reproducibility, efficiency and scalability. All the above software was developed with a minimalist approach in mind and modest funding resources. Yet, it has been proven to be a valuable arsenal for the development and application of key ideas in genome bioinformatics, that supported our own and multiple other research efforts. We hope that the community will embrace these tools and find novel, creative ways of using them.

## References

[1] Ouzounis CA, Coulson RM, Enright AJ, Kunin V, Pereira-Leal JB. Classification schemes for protein structure and function. Nat Rev Genet. 2003;4(7):508–19. doi: 10.1038/nrg1113 . 12838343

[2] Cohen BA, Mitra RD, Hughes JD, Church GM. A computational analysis of whole-genome expression data reveals chromosomal domains of gene expression. Nat Genet. 2000;26(2):183–6. doi: 10.1038/79896 . 11017073

[3] Hinchliff CE, Smith SA, Allman JF, Burleigh JG, Chaudhary R, Coghill LM, et al. Synthesis of phylogeny and taxonomy into a comprehensive tree of life. Proc Natl Acad Sci U S A. 2015;112(41):12764–9. Epub 20150918. doi: 10.1073/pnas.1423041112 ; PubMed Central PMCID: PMC4611642. 26385966 PMC4611642

[4] Kunin V, Cases I, Enright AJ, de Lorenzo V, Ouzounis CA. Myriads of protein families, and still counting. Genome Biol. 2003;4(2):401. Epub 20030128. doi: 10.1186/gb-2003-4-2-401 ; PubMed Central PMCID: PMC151299. 12620116 PMC151299

[5] Rinke C, Schwientek P, Sczyrba A, Ivanova NN, Anderson IJ, Cheng JF, et al. Insights into the phylogeny and coding potential of microbial dark matter. Nature. 2013;499(7459):431–7. Epub 20130714. doi: 10.1038/nature12352 . 23851394

[6] Karp PD, Ouzounis C, Paley S. HinCyc: a knowledge base of the complete genome and metabolic pathways of H. influenzae. Proc Int Conf Intell Syst Mol Biol. 1996;4:116–24. . 8877511

[7] Tsoka S, Ouzounis CA. Recent developments and future directions in computational genomics. FEBS Lett. 2000;480(1):42–8. doi: 10.1016/s0014-5793(00)01776-2 . 10967327

[8] Wren JD, Bateman A. Databases, data tombs and dust in the wind. Bioinformatics. 2008;24(19):2127–8. doi: 10.1093/bioinformatics/btn464 . 18819940

[9] Ouzounis CA. Developing computational biology at meridian 23°E, and a little eastwards. J Biol Res (Thessalon). 2018;25:18. Epub 20181114. doi: 10.1186/s40709-018-0091-5 ; PubMed Central PMCID: PMC6237004. 30460210 PMC6237004

[10] Cole S, Cole JR, Simon GA. Chance and consensus in peer review. Science. 1981;214(4523):881–6. doi: 10.1126/science.7302566 . 7302566

[11] Alberts B, Kirschner MW, Tilghman S, Varmus H. Rescuing US biomedical research from its systemic flaws. Proc Natl Acad Sci U S A. 2014;111(16):5773–7. Epub 20140414. doi: 10.1073/pnas.1404402111 ; PubMed Central PMCID: PMC4000813. 24733905 PMC4000813

[12] Bromham L, Dinnage R, Hua X. Interdisciplinary research has consistently lower funding success. Nature. 2016;534(7609):684–7. doi: 10.1038/nature18315 . 27357795

[13] Harrow J, Drysdale R, Smith A, Repo S, Lanfear J, Blomberg N. ELIXIR: Providing a Sustainable Infrastructure for Life Science Data at European Scale. Bioinformatics. 2021. Epub 20210627. doi: 10.1093/bioinformatics/btab481 ; PubMed Central PMCID: PMC8388016. 34175941 PMC8388016

[14] Smith M, Kunin V, Goldovsky L, Enright AJ, Ouzounis CA. MagicMatch—cross-referencing sequence identifiers across databases. Bioinformatics. 2005;21(16):3429–30. Epub 20050616. doi: 10.1093/bioinformatics/bti548 . 15961438

[15] Cote RG, Jones P, Martens L, Kerrien S, Reisinger F, Lin Q, et al. The Protein Identifier Cross-Referencing (PICR) service: reconciling protein identifiers across multiple source databases. BMC Bioinformatics. 2007;8:401. Epub 20071018. doi: 10.1186/1471-2105-8-401 ; PubMed Central PMCID: PMC2151082. 17945017 PMC2151082

[16] Boeckmann B, Bairoch A, Apweiler R, Blatter MC, Estreicher A, Gasteiger E, et al. The SWISS-PROT protein knowledgebase and its supplement TrEMBL in 2003. Nucleic Acids Res. 2003;31(1):365–70. doi: 10.1093/nar/gkg095 ; PubMed Central PMCID: PMC165542. 12520024 PMC165542

[17] Enright AJ, Kunin V, Ouzounis CA. Protein families and TRIBES in genome sequence space. Nucleic Acids Res. 2003;31(15):4632–8. doi: 10.1093/nar/gkg495 ; PubMed Central PMCID: PMC169885. 12888524 PMC169885

[18] Promponas VJ, Enright AJ, Tsoka S, Kreil DP, Leroy C, Hamodrakas S, et al. CAST: an iterative algorithm for the complexity analysis of sequence tracts. Bioinformatics. 2000;16(10):915–22. doi: 10.1093/bioinformatics/16.10.915 . 11120681

[19] Smith TF, Waterman MS. Identification of common molecular subsequences. J Mol Biol. 1981;147(1):195–7. doi: 10.1016/0022-2836(81)90087-5 . 7265238

[20] Janssen P, Enright AJ, Audit B, Cases I, Goldovsky L, Harte N, et al. COmplete GENome Tracking (COGENT): a flexible data environment for computational genomics. Bioinformatics. 2003;19(11):1451–2. doi: 10.1093/bioinformatics/btg161 . 12874064

[21] Goldovsky L, Janssen P, Ahren D, Audit B, Cases I, Darzentas N, et al. CoGenT++: an extensive and extensible data environment for computational genomics. Bioinformatics. 2005;21(19):3806–10. doi: 10.1093/bioinformatics/bti579 . 16216832

[22] Kunin V, Ahren D, Goldovsky L, Janssen P, Ouzounis CA. Measuring genome conservation across taxa: divided strains and united kingdoms. Nucleic Acids Res. 2005;33(2):616–21. Epub 20050128. doi: 10.1093/nar/gki181 ; PubMed Central PMCID: PMC548337. 15681613 PMC548337

[23] Kunin V, Ouzounis CA. The balance of driving forces during genome evolution in prokaryotes. Genome Res. 2003;13(7):1589–94. doi: 10.1101/gr.1092603 ; PubMed Central PMCID: PMC403731. 12840037 PMC403731

[24] Kunin V, Goldovsky L, Darzentas N, Ouzounis CA. The net of life: reconstructing the microbial phylogenetic network. Genome Res. 2005;15(7):954–9. Epub 20050617. doi: 10.1101/gr.3666505 ; PubMed Central PMCID: PMC1172039. 15965028 PMC1172039

[25] Ouzounis CA, Kunin V, Darzentas N, Goldovsky L. A minimal estimate for the gene content of the last universal common ancestor—exobiology from a terrestrial perspective. Res Microbiol. 2006;157(1):57–68. Epub 20051219. doi: 10.1016/j.resmic.2005.06.015 . 16431085

[26] Sayers EW, Beck J, Bolton EE, Bourexis D, Brister JR, Canese K, et al. Database resources of the National Center for Biotechnology Information. Nucleic Acids Res. 2021;49(D1):D10–D7. doi: 10.1093/nar/gkaa892 ; PubMed Central PMCID: PMC7778943. 33095870 PMC7778943

[27] Cantelli G, Bateman A, Brooksbank C, Petrov AI, Malik-Sheriff RS, Ide-Smith M, et al. The European Bioinformatics Institute (EMBL-EBI) in 2021. Nucleic Acids Res. 2022;50(D1):D11–D9. doi: 10.1093/nar/gkab1127 ; PubMed Central PMCID: PMC8690175. 34850134 PMC8690175

[28] Altschul SF, Gish W, Miller W, Myers EW, Lipman DJ. Basic local alignment search tool. J Mol Biol. 1990;215(3):403–10. doi: 10.1016/S0022-2836(05)80360-2 . 2231712

[29] Buchfink B, Xie C, Huson DH. Fast and sensitive protein alignment using DIAMOND. Nat Methods. 2015;12(1):59–60. Epub 20141117. doi: 10.1038/nmeth.3176 . 25402007

[30] Buchfink B, Reuter K, Drost HG. Sensitive protein alignments at tree-of-life scale using DIAMOND. Nat Methods. 2021;18(4):366–8. Epub 20210407. doi: 10.1038/s41592-021-01101-x ; PubMed Central PMCID: PMC8026399. 33828273 PMC8026399

[31] Enright AJ, Ouzounis CA. BioLayout—an automatic graph layout algorithm for similarity visualization. Bioinformatics. 2001;17(9):853–4. doi: 10.1093/bioinformatics/17.9.853 . 11590107

[32] Goldovsky L, Cases I, Enright AJ, Ouzounis CA. BioLayout(Java): versatile network visualisation of structural and functional relationships. Appl Bioinformatics. 2005;4(1):71–4. doi: 10.2165/00822942-200504010-00009 . 16000016

[33] Freeman TC, Horsewell S, Patir A, Harling-Lee J, Regan T, Shih BB, et al. Graphia: A platform for the graph-based visualisation and analysis of high dimensional data. PLoS Comput Biol. 2022;18(7):e1010310. Epub 20220725. doi: 10.1371/journal.pcbi.1010310 ; PubMed Central PMCID: PMC9352203. 35877685 PMC9352203

[34] Shannon P, Markiel A, Ozier O, Baliga NS, Wang JT, Ramage D, et al. Cytoscape: a software environment for integrated models of biomolecular interaction networks. Genome Res. 2003;13(11):2498–504. doi: 10.1101/gr.1239303 ; PubMed Central PMCID: PMC403769. 14597658 PMC403769

[35] Enright AJ, Van Dongen S, Ouzounis CA. An efficient algorithm for large-scale detection of protein families. Nucleic Acids Res. 2002;30(7):1575–84. doi: 10.1093/nar/30.7.1575 ; PubMed Central PMCID: PMC101833. 11917018 PMC101833

[36] Tatusov RL, Koonin EV, Lipman DJ. A genomic perspective on protein families. Science. 1997;278(5338):631–7. doi: 10.1126/science.278.5338.631 . 9381173

[37] Enright AJ, Ouzounis CA. GeneRAGE: a robust algorithm for sequence clustering and domain detection. Bioinformatics. 2000;16(5):451–7. doi: 10.1093/bioinformatics/16.5.451 . 10871267

[38] Enright AJ, Iliopoulos I, Kyrpides NC, Ouzounis CA. Protein interaction maps for complete genomes based on gene fusion events. Nature. 1999;402(6757):86–90. doi: 10.1038/47056 . 10573422

[39] Iliopoulos I, Enright AJ, Poullet P, Ouzounis CA. Mapping functional associations in the entire genome of Drosophila melanogaster using fusion analysis. Comp Funct Genomics. 2003;4(3):337–41. doi: 10.1002/cfg.287 ; PubMed Central PMCID: PMC2448454. 18629289 PMC2448454

[40] Promponas VJ, Ouzounis CA, Iliopoulos I. Experimental evidence validating the computational inference of functional associations from gene fusion events: a critical survey. Brief Bioinform. 2014;15(3):443–54. Epub 20121205. doi: 10.1093/bib/bbs072 ; PubMed Central PMCID: PMC4017328. 23220349 PMC4017328

[41] Li L, Stoeckert CJ Jr., Roos DS. OrthoMCL: identification of ortholog groups for eukaryotic genomes. Genome Res. 2003;13(9):2178–89. doi: 10.1101/gr.1224503 ; PubMed Central PMCID: PMC403725. 12952885 PMC403725

[42] Page AJ, Cummins CA, Hunt M, Wong VK, Reuter S, Holden MT, et al. Roary: rapid large-scale prokaryote pan genome analysis. Bioinformatics. 2015;31(22):3691–3. Epub 20150720. doi: 10.1093/bioinformatics/btv421 ; PubMed Central PMCID: PMC4817141. 26198102 PMC4817141

[43] Wright DW, Angus T, Enright AJ, Freeman TC. Visualisation of BioPAX Networks using BioLayout Express (3D). F1000Res. 2014;3:246. Epub 20141020. doi: 10.12688/f1000research.5499.1 ; PubMed Central PMCID: PMC4406191. 25949802 PMC4406191

